# Characterizing sociodemographic disparities and predictors of Gestational Diabetes Mellitus among Asian and Native Hawaiian or other Pacific Islander pregnant people: an analysis of PRAMS data, 2016–2022

**DOI:** 10.1186/s12884-024-07034-5

**Published:** 2024-12-20

**Authors:** Mallory Go, Natasha Sokol, L. G. Ward, Micheline Anderson, Shufang Sun

**Affiliations:** 1 Department of Epidemiology, Brown University School of Public Health, Providence, RI USA; 2 Department of Psychiatry and Human Behavior, Brown University Warren Alpert Medical School, Providence, RI USA; 3 Center for Behavioral and Preventive Medicine, The Miriam Hospital, Providence, RI USA; 4 Department of Behavioral and Social Sciences, Brown University School of Public Health, Providence, RI USA; 5 International Health Institute, Brown University School of Public Health, Providence, RI USA; 6 Mindfulness Center, Brown University School of Public Health, Providence, RI USA

**Keywords:** Gestational Diabetes Mellitus, PRAMS, Social determinants of health, Asian and NHOPI, Pregnancy

## Abstract

**Background:**

Gestational Diabetes Mellitus (GDM) affects between 2 and 10% of pregnancies in the United States, with trends of increasing prevalence and a significant amount of variability across race and ethnicity, maternal age, and insurance status. Asian and Native Hawaiian or Other Pacific Islanders (NHOPI) have been documented to have a higher prevalence and risk of developing GDM compared to non-Hispanic white populations and have been under-studied in health disparities research.

**Methods:**

Using data from the Pregnancy Risk Assessment Monitoring System (PRAMS) 2016–2022 surveys, we conducted analyses for the overall PRAMS sample as well as within-group analyses among participants who identify as Asian and NHOPI to identify risk factors for GDM. Descriptive statistics were also collected in the Asian and NHOPI subsample, stratified by Asian and NHOPI ethnicity. Bivariate analyses were performed to explore the relationship between potential GDM risk factors among the overall analytic sample and within the Asian and NHOPI subsample, and multivariable logistic regression was used to investigate potential predictors of GDM.

**Results:**

Asian and NHOPI ethnicities differed by prevalence of GDM at 17.2%, 19.56%, 10.8%, 10.71%, and 18.49% for Chinese, Filipino, Japanese, Native Hawaiian/Other Pacific Islander, and Other Asian, respectively. Compared to White individuals (reference group), the odds of GDM were higher for Asian and Native Hawaiian/Other Pacific Islander individuals in the adjusted model (adjusted odds ratio (aOR) = 2.19, 95% CI: 2.62–2.9). Native mothers also demonstrated significantly elevated odds (aOR = 1.48, 95% CI: 1.4–1.6), while Mixed-race individuals exhibited slightly increased odds (OR = 1.22, 95% CI: 1.14–1.29). The findings revealed notable variability in GDM risk factors across ANHOPI subgroups. Obesity emerged as a consistent and strong predictor of GDM across all groups, while other factors such as interpersonal violence exposure and prenatal depression demonstrated limited or subgroup specific effects.

**Conclusion:**

This analysis of 2016 to 2022 PRAMS data illustrated significant variations of GDM predictors between the general population and the Asian and NHOPI population, as well as differences between Asian and NHOPI ethnicities.

## Background

Gestational diabetes mellitus (GDM), one of the most common pregnancy complications, is defined as glucose intolerance that develops or is first recognized during pregnancy (Hunsberger et al., 2010). GDM affects between 2 and 10% of pregnancies in the United States, and its prevalence is increasing over time [1, 2]. There is a significant amount of variability in GDM prevalence between US states, which is attributed to compositional differences in race and ethnicity, maternal age, insurance status, obesity, income, and hospital factors (type and bed count) [3, 4].

The health and economic burden of GDM is over $1.8 billion annually, and includes adverse maternal and child health outcomes beyond pregnancy including, but not limited to, progression to Type 2 diabetes mellitus, macrosomia and associated delivery complications, increased risk of maternal mortality, and increased risk of developing metabolic syndrome in childhood [5]. While 70–85% of pregnant people diagnosed can manage GDM via lifestyle adjustments, the COVID-19 pandemic adversely impacted GDM [6]. GDM-related stress, depression, and anxiety can be a barrier to forming and maintaining healthy habits through the postpartum period, which is essential to reducing the risk of T2DM and other adverse health outcomes [7].

Asian, NHOPI, Native American, African American, and Hispanic populations have been documented to have a higher prevalence of GDM compared to non-Hispanic white populations, with indications that Asians, persons having origins in the peoples of East Asia, Southeast Asia, and India, shoulder the greatest risk [8, 9]. Historically, Asian and NHOPI peoples have been aggregated as a homogenous group in national surveys and studies, despite distinct culture, language, and health behavior practices, or excluded due to small sample sizes [10, 11], and existing studies on GDM in this population have generally presented findings from aggregated data [12–16]. Asian and NHOPI is a heterogenous group that represents over 50 distinct ethnicities with distinct cultures and experiences in the US [17, 18]. The largest Asian and NHOPI ethnicity groups – Chinese, Filipino, Asian Indian, Vietnamese, Korean, and Japanese – represent 87% of Asian and NHOPI in the US and even between these main groups, there are significant cultural differences such as language, diet, and social norms [19, 20]. Therefore, it is crucial to conduct research with disaggregated data to accurately capture the diverse experiences and needs of each ethnicity within the Asian and NHOPI populations.

Psychosocial factors such as prenatal depression, anxiety, and experiences of interpersonal violence (IPV) were included in the analyses because of their significant impact on maternal health and pregnancy outcomes. For Asian and Native Hawaiian/Other Pacific Islander (Asian and NHOPI) populations, these psychosocial factors may uniquely influence GDM risk and management due to the distinct cultural, social, and economic stressors faced by this group [21–24]. Although Asian and NHOPI individuals are documented to have a higher prevalence of GDM compared to non-Hispanic white populations, psychosocial aspects related to pregnancy health are often underexplored in this group [9]. Additionally, aggregated data on Asian and NHOPI populations has often overlooked the diversity within this group, resulting in insufficient understanding of how specific psychosocial stressors might interact with cultural norms or healthcare access issues [9, 25]. Research suggests that culturally distinct norms surrounding social support, mental health stigma, and familial roles may shape how Asian and NHOPI women experience and manage GDM and related psychosocial stressors [26–28]. Therefore, investigating prenatal depression, anxiety, and IPV in Asian and NHOPI populations is essential for identifying unique psychosocial barriers and developing culturally tailored interventions that address both the mental and physical health needs of these diverse groups.

As of 2019, 67% of Health and Human Services (HHS) surveys collected disaggregated data on Asian and NHOPI people, meaning that the remaining 33%, much like other federal health data collection methods, only collected aggregated data [12]. This aggregation combined with the persistent “model minority myth” – that all Asian and NHOPIs experience academic, occupational, and financial success, and are generally healthier than Whites and other minorities, obscures differences in maternal child health and hinders progress in eliminating these disparities [12]. For example, a National Vital Statistics System (NVSS) report compared neonatal death rates (per 1,000 live births) for 2018 among all mothers, aggregated Asian and NHOPI (including Pacific Islanders) mothers, and Native Hawaiian/Pacific Islander mothers (disaggregated from the Asian and NHOPI population) at 3.8, 2.8, and 5.3, respectively, demonstrating significant hidden disparity between aggregated and disaggregated data [14, 29].

Despite the recognition of higher GDM prevalence among Asian and NHOPI populations, there remains a significant gap in the literature in understanding GDM risk and outcomes across different Asian and NHOPI ethnicities [30]. Most studies have either aggregated Asian and NHOPI data, thereby masking intra-group differences, or have excluded Asian and NHOPI subgroups due to small sample sizes, limiting the generalizability and applicability of the findings [10, 11]. Thus, it is important to conduct research that utilizes data disaggregated by specific Asian and NHOPI ethnicities, thereby providing a more granular understanding of GDM prevalence and risk factors within these groups to provide critical insights into the heterogeneous nature of the Asian and NHOPI community in the context of GDM [18–20]. Where the literature cited focused on a certain population, (i.e. Asian American, Pacific Islander, Southeast Asian, etc.), which specific population it is referring to will be referenced as it was in the original literature.

In response to this issue, our study conducted two separate analyses. The first model assessed GDM disparities in the general population, categorizing race and ethnicity into broad groups, including American Indian or Alaska Native, Black, white, and Asian and NHOPI, with white individuals as the reference group. To address the limitations of aggregation, the second model focused specifically on the Asian and NHOPI population, further disaggregating these groups into categories such as Chinese, Japanese, Filipino, Native Hawaiian, and other Asian identities. This approach underscores the importance of understanding GDM risk within disaggregated Asian and NHOPI subpopulations without implying a hierarchy of importance among these groups, and it allows for more nuanced insights that can guide culturally and contextually relevant health interventions.

The current project uses a national dataset on maternal and fetal health collected by the Centers for Disease Control and Prevention (CDC) to (a) estimate racial and social disparities in gestational diabetes mellitus in the most recent survey, and (b) conduct within group analysis to examine GDM, focusing on Asian and Native Hawaiian/Other Pacific Islanders (Asian and NHOPI) to identify risk factors within disaggregated subpopulations.

## Methods

The current project used data from The CDC’s National Pregnancy Risk Assessment Monitoring System (PRAMS) Phase 8, a nationally representative survey on maternal and fetal health conducted by the CDC between 2016 and 2022. Phase 8 data was used as it was the most recent phase that included data for Hawaii, where Asian and NHOPI individuals make up a significant proportion of the state population [31].

### The PRAMS dataset

PRAMS is a national surveillance system that provides data about pregnancy and the first few months after birth, maternal health indicators, and pregnancy outcomes of interest used to assess the health of mothers with the goals of improving maternal and infant health outcomes. PRAMS represents approximately 83% of all US live births, and over-samples minority groups and those who delivered low-birth-weight infants. Specific birth certificate variables are aggregated to protect participant confidentiality such as maternal age and geographic indicators [17, 18]. Specifically, we analyzed data from PRAMS Phase 8 (2016–2022), including core questionnaire data, standard questionnaire data, and birth certificate variables.

### Measures

#### Outcome

The primary outcome of interest was the diagnosis of GDM. GDM was a binary variable assessed in PRAMS using the prompt, “*During your most recent pregnancy, did you have any of the following health conditions?*” Under this question, participants were considered to have had GDM if they checked “Yes” for “Gestational diabetes (diabetes that started during this pregnancy).”

#### Race and ethnicity

Two separate models were conducted. In one model for the general population, race was grouped into the following exclusive categories: American Indian or Alaska Native, African American (“Black”), white, and Asian and Native Hawaiian or Other Pacific Islander (“Asian and NHOPI”). Ethnicity was defined into exclusive categories: “Hispanic” or “Non-Hispanic”. For the general population analyses, white individuals were the reference group.

To further estimate differences between Asian and NHOPI ethnicities, another model only consisting of the Asian and NHOPI population was conducted. Ethnicities under the “Asian and NHOPI” category was defined into exclusive categories: “Chinese”, “Japanese”, “Filipino”, “Native Hawaiian”, and “Other Asian” for the disaggregated Asian and NHOPI sample. Native Hawaiian individuals were selected as the reference group for the Asian and NHOPI model analyses due to their distinct cultural, historical, and social contexts, as well as their documented health disparities compared to other Asian and NHOPI subgroups; this choice allows for more meaningful comparisons within the Asian and NHOPI population [15, 16, 30].

##### Other demographic variables

Maternal age was originally a categorical variable with seven groups (< = 17, 18–19, 20–24, 25–29, 30–34, 35–39, and 40 +). Based on the literature on the potential association between maternal age and GDM, groups were combined to yield the binary variable with categories of < 25 years of age and ≥ 25 years of age [32, 33]. Due to lack of standardization across states, education was only available as a categorical variable of years of education (0–8 years, 9–11 years, 12 years, 13–15 years, ≥ 16 years. As a result, maternal education was divided into three categories: high school education or less (0–12 years), some college (13–15 years) and college graduate or greater ($\ge$ 16 years). Prenatal body mass index (BMI) was categorized into underweight (< 18.5), normal weight (18.5 to < 25.0), overweight (25.0 to < 30), and obese (> 30.0) [34, 35]. Insurance status fell into one of three groups: public (Medicaid, SCHIP/CHIP, other), private (Employment-based, Healthcare exchange, parent’s insurance, military-specific, IHS), or uninsured.

##### Psychosocial and behavioral health factors

Binary variables chosen based on existing literature on GDM risk factors were used to capture conditions diagnosed or reported around the time of pregnancy (before or during) [36–38]. Prenatal depression and anxiety were determined by binary questions regarding any health conditions the pregnant person might have had during the three months before the pregnancy began. Respondents were considered to have smoked if they had greater than zero cigarettes in the three months before pregnancy. Respondents were considered to have experienced interpersonal violence (IPV) if they answered “Yes” to the question, “In the 12 months before you got pregnant with your new baby, did any of the following people push, hit, slap, kick, choke, or physically hurt you in any other way?”. Women, Infants, Children (WIC) or Special Supplemental Nutrition Program (SSNP) use was determined based on the question, “During your most recent pregnancy, were you on WIC (the Special Supplemental Nutrition Program for Women, Infants, and Children)?”.

### Analytic sample

The original sample consisted of 240,724 individuals in the US who completed the PRAMS survey in 2016 to 2022 [39]. Those who reported having “Type 1 or Type 2 diabetes (not gestational diabetes or diabetes that starts during pregnancy)” were excluded from the analytic sample, due to the potential of confounding from entering pregnancy with pre-existing disorders of glucose metabolism [40]. Those with missing information on GDM or maternal race were also excluded from the analytic sample. Upon examination of this sample, missingness on all other variables was found to be approximately 5%, and therefore complete case analysis was used [41, 42]. After eliminating all respondents with missing data on any of the variables under examination, the final analytic sample consisted of *N* = 197,236 subjects with complete data.

**Fig. 1:**
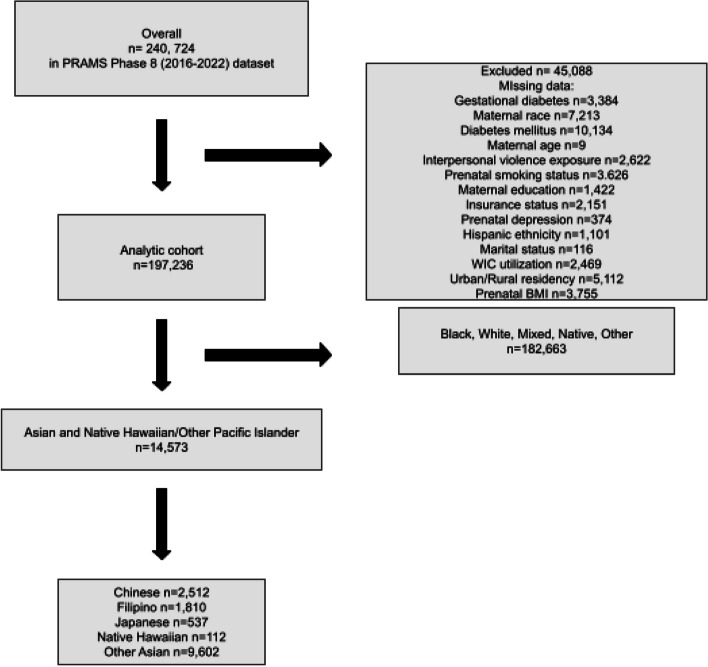
Analytic sample flow chart

For the Asian and NHOPI subsample, those who did not self-report Asian race or that had missing information for the maternal Asian and NHOPI race/ethnicity birth certificate variable were excluded from the Asian and NHOPI subsample. The final Asian and NHOPI subsample consisted of *N* = 14,573 subjects who had complete, valid data for GDM and maternal Asian and NHOPI ethnicity (see Fig. 1).

### Statistical analysis

Data were cleaned, entered, and analyzed using STATA/S.E. Version 17.0. [43]. Pearson’s chi-square test was used to approximate the association between the sociodemographic variables of interest and potential covariates. All analyses included weights provided by PRAMS to account for the greater probability of inclusion for some individuals due to oversampling and survey design [44].

Descriptive analysis was performed within the entire analytic sample and within the Asian and NHOPI subsample for all potential predictors by GDM status. The bivariate analysis was used first to explore correlation, and the results of the analysis informed the multivariable analysis [45, 46]. Dummy coding was used for categorical variables in non-dichotomous questions.

Binary logistic regression estimated the crude odds ratios, with associated 95% confidence intervals and *p*-values between potential predictors and GDM within the overall population, then specifically the Asian and NHOPI subsample. A correlation screen and multicollinearity screen were used to identify potential collinear variables with a correlation coefficient of ± 0.5 as the threshold for moderate association [47]. The bivariate analysis results were used to identify potential predictors that were significantly associated with GDM [48–50]. Multivariable logistic regression was applied to measure adjusted odds ratios (aOR). In reporting the adjusted odds ratios (aOR) compared to the crude odds ratios (OR), variables included in the final multivariable logistic regression model were determined using directed acyclic graphs (DAGs) and causal inference framework. The crude and adjusted models were evaluated using the Bayesian Information Criterion (BIC) to determine the most parsimonious model. A likelihood ratio test (LRT) was used to compare the crude and adjusted models and resulted in a nested model for the overall analytic sample and the Asian and NHOPI subsample, separately.

## Results

### Sample characteristics

Table 1 provided demographic and clinical characteristics of the PRAMS analytic sample stratified by gestational diabetes mellitus (GDM) status. Of the total sample (*N* = 197,236), 10.7% of individuals reported a diagnosis of GDM. Individuals identifying as Native Hawaiian/Other Pacific Islander had the highest prevalence of GDM (18.05%), followed by Native American/Alaskan Native (12.97%), and those identifying as multiracial (11.0%). White individuals had the lowest prevalence of GDM (9.14%). Similarly, a greater proportion of non-Hispanic individuals were diagnosed with GDM compared to Hispanic individuals (11.93% vs. 9.85%, respectively; *p* < 0.001). The prevalence of GDM was significantly higher among individuals aged ≥ 25 years compared to those aged < 25 years (11.5% vs. 5.48%; *p* < 0.001). The distribution of BMI categories varied significantly between GDM and non-GDM groups. Obesity was overrepresented in the GDM group, with 16.35% of individuals having a BMI ≥ 30 compared to 6.29% with a normal BMI (18.5 to < 25.0) (*p* < 0.001). A lower proportion of uninsured individuals (9768%) had GDM compared to individuals with private insurance (10.18%) or uninsured individuals (11.28%; *p* < 0.001). Urban-dwelling individuals were slightly more likely to be diagnosed with GDM compared to those in rural areas (10.27% vs. 9.79%; *p* = 0.004). GDM was more prevalent among individuals with prenatal depression (11.2%) compared to those without prenatal depression (10.01%; *p* < 0.001). GDM prevalence also varied by education level, with the highest prevalence among those with some college education (10.7%) compared to those with high school or less (10.03%) and those with college or greater (9.89%; *p* < 0.001). Individuals who utilized WIC services had a slightly higher prevalence of GDM compared to those who did not (10.47% vs. 10.0%; *p* = 0.001). No significant differences were observed in the distribution of interpersonal violence exposure (*p* = 0.073) or prenatal smoking status (*p* = 0.837) between the GDM and non-GDM groups.

**Table 1:** Descriptive statistics of analytic sample

	No GDM (*n* = 177,176, 89.83%)		GDM (*n* = 20,060, 10.17%)		
	n	Freq. (%)	n	Freq. (%)	*p*-value
**Maternal race**					< 0.001
Asian and Native Hawaiian/Other Pacific Islander	11,942	81.95	2631	18.05	
Black	33,856	91.13	3296	8.87	
Mixed	10,831	89	1338	11	
Native	7577	87.03	1129	12.97	
Other	9011	88.15	1211	11.85	
White	103,959	90.86	10,455	9.14	
**Hispanic ethnicity**					< 0.001
Hispanic	27,087	88.07	3670	11.93	
Non-Hispanic	150,089	90.15	16,390	9.85	
**Maternal age**					< 0.001
< 25	41,109	94.52	2384	5.48	
> = 25	136,067	88.5	17,676	11.5	
**Maternal pre-pregnancy BMI**					< 0.001
Underweight (< 18.5)	5770	95.2	291	4.8	
Normal weight (18.5 to < 25.0)	76,816	93.71	5160	6.29	
Overweight (25.0 to < 30)	46,552	89.91	5222	10.09	
Obese (> 30.0)	48,038	83.65	9387	16.35	
**Martial status**					< 0.001
Married	106,474	89.14	12,977	10.86	
Not married	70,702	90.89	7083	9.11	
**Insurance status**					< 0.001
Public	59,078	90.24	6393	9.76	
Private	97,798	89.82	11,086	10.18	
Uninsured	20,300	88.72	2581	11.28	
**Residence**					0.004
Rural	36,806	90.21	3993	9.79	
Urban	140,370	89.73	16,067	10.27	
**Interpersonal violence**					0.073
Exposure to violence	7023	90.43	743	9.57	
No exposure to violence	170,153	89.8	19,317	10.2	
**Prenatal depression**					< 0.001
Prenatal depression	24,718	88.88	3094	11.12	
No prenatal depression	152,458	89.99	16,966	10.01	
**Prenatal smoking status**					0.837
Smoking	33,739	89.8	3832	10.2	
No smoking	143,437	89.84	16,228	10.16	
**Maternal education**					< 0.001
High school or less	62,552	89.97	6975	10.03	
Some college	50,162	89.3	6012	10.7	
College or greater	64,462	90.11	7073	9.89	
**WIC utilization**					0.001
WIC	62,691	89.53	7335	10.47	
No WIC	114,485	90	12,725	10	

Table 2 presented descriptive statistics stratified by race and ethnicity among the general PRAMS sample (*N* = 197,236), with significant differences observed across several sociodemographic, clinical, and behavioral factors. Maternal age was significantly associated with race and ethnicity (*p* < 0.001). Notably, individuals identifying as Asian and Native Hawaiian/Other Pacific Islander had the highest percentage of mothers aged ≥ 25 years (92.25%), while Native individuals had the highest percentage of younger mothers (< 25 years, 36.55%%). Pre-pregnancy BMI distributions also varied significantly across racial and ethnic groups (*p* < 0.001). Obesity (BMI > 30) was most prevalent among Black individuals (40.07%), while Asian and Native Hawaiian/Other Pacific Islander individuals had the highest proportion of individuals in the normal weight category (58.07%). Insurance type varied significantly across racial and ethnic groups (*p* < 0.001). Individuals identifying as Native had the highest proportion of public insurance use (79.18%), whereas Asian and Native Hawaiian/Other Pacific Islander individuals had the lowest proportion of public insurance use (18.4%). Uninsured rates were highest among Other racial and ethnic groups (37.49%) and lowest among Asian and Native Hawaiian/Other Pacific Islander individuals (6.66%). Interpersonal violence exposure differed significantly by race and ethnicity (*p* < 0.001). Mixed individuals reported the highest prevalence of interpersonal violence exposure (9.43%), whereas Asian and Native Hawaiian/Other Pacific Islander individuals had the lowest prevalence (1.23%). The prevalence of prenatal depression also varied significantly across racial and ethnic groups (*p* < 0.001). Mixed individuals reported the highest prevalence (20.51%) and Asian and Native Hawaiian/Other Pacific Islander individuals reported the lowest prevalence (3.33%). Asian and Native Hawaiian/Other Pacific Islander individuals had the highest proportion of those with a college education or greater (66.03%), while Native individuals had the lowest proportion (9.48%).

**Table 2:** Descriptive statistics by maternal race

	Asian and Native Hawaiian/Other Pacific Islander (*n* = 14,573, 7.39%)		Black (*n* = 37,152, 18.84%)		Mixed (*n* = 12,168, 6.17%)		Native (*n* = 8706, 4.41%)		Other (*n* = 10,222, 5.18%)		White (*n* = 114,414, 58.01%)		
	n	Freq. (%)	n	Freq. (%)	n	Freq. (%)	n	Freq. (%)	n	Freq. (%)	n	Freq. (%)	*p*-value
**Maternal age**													< 0.001
< 25	1130	7.75	10,703	28.81	3559	29.25	3182	36.55	2826	27.65	22,093	19.31	
> = 25	13,443	92.25	26,449	71.19	8610	70.75	5524	63.45	7396	72.35	92,321	80.69	
**Maternal pre-pregnancy BMI**													< 0.001
Underweight (< 18.5)	862	5.92	1052	2.83	352	2.89	152	1.75	240	2.35	3403	2.97	
Normal weight (18.5 to < 25.0)	8463	58.07	11,436	30.78	4612	37.9	2695	30.96	3845	37.61	50,925	44.51	
Overweight (25.0 to < 30)	3417	23.45	9779	26.32	3136	25.77	2394	27.5	3290	32.19	29,758	26.011	
Obese (> 30.0)	1831	12.56	14,885	40.07	4069	33.44	3465	39.8	2847	27.85	30,328	26.51	
**Martial status**													< 0.001
Married	12,613	86.55	11,879	31.97	6004	49.34	2642	30.35	5132	50.21	81,181	70.95	
Not married	1960	13.45	25,273	68.03	6165	50.66	6064	69.65	5090	49.79	33,233	29.05	
**Insurance status**													< 0.001
Public	2681	18.4	19,367	52.13	5588	45.92	6893	79.18	3002	29.37	27,940	24.42	
Private	10,922	74.95	13,874	37.34	5490	45.11	1190	13.67	3388	33.14	74,020	64.69	
Uninsured	970	6.66	3911	10.53	1091	8.97	623	7.16	3832	37.49	12,454	10.89	
**Residence**													< 0.001
Rural	990	6.79	3644	9.81	2790	22.93	5163	59.3	1400	13.7	26,812	23.43	
Urban	13,583	93.21	33,508	90.19	9379	77.07	3543	40.7	8822	86.3	87,602	76.57	
**Interpersonal violence**													< 0.001
Exposure to violence	179	1.23	1904	5.12	843	6.93	821	9.43	332	3.25	3687	3.22	
No exposure to violence	14,394	98.77	35,248	94.88	11,326	93.07	7885	90.57	9890	96.75	110,727	96.78	
**Prenatal depression**													< 0.001
Prenatal depression	486	3.33	4773	12.85	2496	20.51	1784	20.49	798	7.81	17,475	15.27	
No prenatal depression	14,087	96.67	32,379	87.15	9673	79.49	6922	79.51	9424	92.19	96,939	84.73	
**Prenatal smoking status**													< 0.001
Smoking	713	4.89	6341	17.07	3383	27.8	3801	43.66	776	7.59	22,557	19.72	
No smoking	13,860	95.11	30,811	82.93	8786	72.2	4905	56.34	9446	92.41	91,857	80.28	
**Maternal education**													< 0.001
High school or less	2611	17.92	16,872	45.41	4493	36.92	5005	57.49	6555	64.13	33,991	29.71	
Some college	2340	16.02	13,088	35.23	4297	35.31	2876	33.03	2222	21.74	31,351	27.4	
College or greater	9622	66.03	7192	19.36	3379	27.77	825	9.48	1445	14.14	49,072	42.89	
**WIC utilization**													< 0.001
WIC	3037	20.84	20,840	56.09	4934	40.55	5070	58.24	5892	57.64	30,253	26.44	
No WIC	11,536	79.16	16,312	43.91	7235	59.45	3636	41.76	4330	42.36	84,161	73.56	

Table 3 represents the descriptive statistics of 14,573 Asian and Native Hawaiian/Other Pacific Islander (ANHOPI) individuals. Within the ANHOPI group, the majority identified as “Other Asian” (65.91%), followed by Chinese (17.17%), Filipino (12.45%), Japanese (3.69%), and Native Hawaiian (0.77%). The prevalence of GDM differed significantly across ANHOPI subgroups (*p* < 0.001). Among those with GDM, “Other Asian” individuals constituted the largest proportion (65.89%), followed by Chinese (17.24%), Filipino (12.42%), Japanese (3.68%), and Native Hawaiian (0.77%). A higher maternal age (≥ 25 years) was observed among most individuals across all subgroups (*p* < 0.001). Japanese individuals had the highest proportion in this category (99.26%), while younger maternal age (< 25 years) was more prevalent among Native Hawaiian/Other Pacific Islanders (19.64%). BMI distribution varied significantly across subgroups (*p* < 0.001). Obesity (BMI > 30) was most prevalent among Native Hawaiian/Other Pacific Islander individuals. Exposure to interpersonal violence (IPV) and prenatal depression showed significant subgroup differences. More Native Hawaiian/Other Pacific Islanders reported exposure to IPV (8.93%) compared to Japanese individuals (0.56%). Prenatal depression was highest among Native Hawaiian/Other Pacific Islanders (14.29%) compared to Chinese individuals (2.27%) (*p* < 0.001).

**Table 3:** Descriptive statistics by Asian and Native Hawaiian/Other Pacific Islander Ethnicity

	Chinese (*n* = 2512, 17.24%)		Filipino (*n* = 1810, 12.42%)		Japanese (*n* = 537, 3.68%)		Native Hawaiian (*n* = 112, 0.77%)		Other Asian (*n* = 9602, 65.89%)		
	n	Freq. (%)	n	Freq. (%)	n	Freq. (%)	n	Freq. (%)	n	Freq. (%)	*p*-value
**GDM status**											< 0.001
Gestational diabetes	432	17.2	354	19.56	58	10.8	12	10.71	1775	18.49	
No gestational diabetes	2080	82.8	1456	80.44	479	89.2	100	89.29	7827	81.51	
**Maternal age**											< 0.001
< 25	76	3.03	158	8.73	4	0.74	22	19.64	870	9.06	
> = 25	2436	96.97	1652	91.27	533	99.26	90	80.36	8732	90.94	
**Maternal pre-pregnancy BMI**											< 0.001
Underweight (< 18.5)	267	10.63	69	3.81	53	9.87	5	4.46	468	4.87	
Normal weight (18.5 to < 25.0)	1873	74.56	955	52.76	365	67.97	29	25.89	5241	54.58	
Overweight (25.0 to < 30)	285	11.35	490	27.07	82	15.27	24	21.43	2536	26.41	
Obese (> 30.0)	87	3.46	296	16.35	37	6.89	54	48.21	1357	14.13	
**Martial status**											< 0.001
Married	2283	90.88	1475	81.49	484	90.13	65	58.04	8306	86.5	
Not married	229	9.12	335	18.51	53	9.87	47	41.96	1296	13.5	
**Insurance status**											< 0.001
Public	403	16.04	377	20.83	61	11.36	54	48.21	1786	18.6	
Private	1981	78.86	1324	73.15	453	84.36	47	41.96	7117	74.12	
Uninsured	128	5.1	109	6.02	23	4.28	11	9.82	699	7.28	
**Residence**											< 0.001
Rural	92	3.66	325	17.96	76	14.15	33	29.46	464	4.83	
Urban	2420	96.34	1485	82.04	461	85.85	79	70.54	9138	95.17	
**Interpersonal violence**											< 0.001
Exposure to violence	20	0.8	31	1.71	3	0.56	10	8.93	115	1.2	
No exposure to violence	2492	99.2	1779	98.29	534	999.44	102	91.07	9487	98.8	
**Prenatal depression**											< 0.001
Prenatal depression	57	2.27	93	5.14	18	3.35	16	14.29	302	3.15	
No prenatal depression	2455	97.73	1717	94.86	519	96.65	96	85.71	9300	96.85	
**Prenatal smoking status**											< 0.001
Smoking	60	2.39	134	7.4	24	4.47	28	25	467	4.86	
No smoking	2452	97.61	1676	92.6	513	95.53	84	75	9135	95.14	
**Maternal education**											< 0.001
High school or less	298	11.86	290	16.02	33	6.15	56	50	1934	20.14	
Some college	301	11.98	496	27.4	118	21.97	35	31.25	1390	14.48	
College or greater	1913	76.15	1024	56.57	386	71.88	21	18.75	6278	65.38	
**WIC utilization**											< 0.001
WIC	490	19.51	378	20.88	47	8.75	49	43.75	2073	21.59	
No WIC	2022	80.49	1432	79.12	490	91.25	63	56.25	7529	78.41	

### Social determinants of health and GDM Status

Table 4 represents the multivariable logistic regression model depicting the association of social determinants of health and the risk of GDM for the entire analytic sample. Compared to White individuals (reference group), the odds of GDM were higher for Asian and Native Hawaiian/Other Pacific Islander individuals in the adjusted model (adjusted odds ratio (aOR) = 2.19, 95% CI: 2.62–2.9). Native mothers also demonstrated significantly elevated odds (aOR = 1.48, 95% CI: 1.4–1.6), while Mixed-race individuals exhibited slightly increased odds (OR = 1.22, 95% CI: 1.14–1.29). Individuals with an obese BMI had more than three times the odds of GDM compared to those with normal BMI (aOR = 3.23, 95% CI: 3.1–3.35). Overweight mothers also experienced increased odds (aOR = 1.73, 95% CI: 1.66–1.8). Exposure to interpersonal violence (IPV) during pregnancy did not significantly predict adverse outcomes in either unadjusted or adjusted models. However, prenatal depression was a significant predictor of GDM. Individuals with prenatal depression had 21% higher odds of GDM compared to those without depression in the adjusted model (OR = 1.21, 95% CI: 1.16–1.26). This association was consistent across all models.

**Table 4:** General odds ratios

	**Unadjusted model**^**1**^	**Adjusted model**^**2**^	**Adjusted model**^**3**^	**Adjusted model**^**4**^	**BIC**
	**OR (95% CI)**	**OR (95% CI)**	**OR (95% CI)**	**OR (95% CI)**	
**Race & ethnicity**					122,735.7*
Asian/Native Hawaiian or Other Pacific Islander	2.19 (2.09, 2.3)	2.76 (2.62, 2.9)			
Black	0.97 (0.93, 1.01)	0.92 (0.87, 0.96)			
Mixed	1.23 (1.16, 1.3)	1.22 (1.14, 1.29)			
Native	1.48 (1.39, 1.58)	1.48 (1.4, 1.6)			
Other	1.34 (1.25, 1.42)	1.08 (1.0, 1.16)			
White	ref	ref	ref	ref	
**Prenatal BMI**					122,801.3*
Underweight	0.75 (0.67, 0.85)		0.78 (0.69, 0.88)		
Normal	ref	ref	ref	ref	
Overweight	1.67 (1.6, 1.74)		1.73 (1.66, 1.8)		
Obese	2.91 (2.81, 3.02)		3.23 (3.1, 3.35)		
**Interpersonal violence exposure**					128,496.1*
No exposure	ref	ref	ref	ref	
Exposure	0.93 (0.86, 1.01)			0.95 (0.88, 1.02)	
**Prenatal depression**					128,419.5*
No depression	ref	ref	ref	ref	
Depression	1.12 (1.08, 1.17)			1.21 (1.16, 1.26)	
**Insurance status**					128,482.2*
Private	ref	ref	ref	ref	
Public	0.95 (0.92, 0.99)			0.94 (0.91, 0.97)	
Uninsured	1.12 (1.08, 1.17)			1.07 (1.02, 1.12)	

*OR* odds ratio, *CI* confidence interval

^1^Unadjusted for confounders

^2^Adjusted for maternal race, maternal ethnicity, maternal age, education, insurance status, WIC utilization, smoking status, prenatal BMI, prenatal depression, exposure to IPV, urban/rural residency

^3^Adjusted for maternal race, maternal ethnicity, insurance status, maternal age

^4^Adjusted for adjusted for maternal race, maternal ethnicity

^*^Lowest BIC presented is the adjusted model

^a^Insufficient cell count

### Social determinants of health and GDM risk among Asian and NHOPI individuals

Tables 5, 6, 7, 8 and 9 represents the multivariable logistic regression model depicting the association of social determinants of health and the risk of GDM exclusively within people who identify as Asian and NHOPI. Higher BMI categories were significantly associated with increased adjusted odds of GDM in Chinese individuals (Table 5). Individuals with an obese BMI (> 30.0) had 1.84 times the odds of GDM (95% CI: 1.13–2.99) compared to women with normal BMI (Table 5). Regarding interpersonal violence (IPV) exposure, prenatal depression, and insurance status, no significant associations with GDM were observed (Table 5). Filipino individuals with an obese BMI had 2.17 times the odds of GDM (95% CI: 1.59–2.97) compared to those with normal BMI in the adjusted model (Table 6). Similarly to Chinese individuals, IPV exposure, prenatal depression, and insurance status were not significantly associated with GDM (Table 6). Japanese individuals had the smallest sample size for maternal age out of the Asian and NHOPI ethnicities and consequently models did not have sufficient observations to adjust for maternal age and insurance status categories had insufficient cell counts for multivariable logistic regression modeling (Table 7). Japanese individuals classified as obese exhibited markedly higher odds of GDM (aOR = 9.06, 95% CI: 4.24–19.4), though this was not statistically significant due to wide confidence intervals (Table 7). No significant association was observed between GDM and IPV exposure or prenatal depression within the Japanese population (Table 7). In the Native Hawaiian/Other Pacific Islander population, individuals classified as obese exhibited higher odds of GDM (adjusted OR = 3.01, 95% CI: 0.57–15.92), though this was not statistically significant due to wide confidence intervals (Table 8). No significant association was observed between GDM and IPV exposure, prenatal depression, or insurance status (Table 8). Finally, in the Other Asian population, obesity was strongly associated with GDM, with an adjusted OR of 2.12 (95% CI: 1.84, 2.45) (Table 9). No significant relationship between IPV exposure and GDM was observed (aOR = 1.04, 95% CI: 0.65–1.67) (Table 9). Prenatal depression was significantly associated with higher GDM odds in both models, with a stronger effect in the adjusted model (aOR = 1.47, 95% CI: 1.12–1.94) compared to the crude model (OR = 1.34, 95% CI: 1.02–1.76) (Table 9). Public (aOR = 0.97, 95% CI: 0.84–1.12) and private insurance status (aOR = 1.11, 95% CI: 0.91–1.36) were not significantly associated with increased odds of GDM (Table 9).

**Table 5:** AANHOPI odds ratios: Chinese

	**Unadjusted model**^**1**^	**Adjusted model**^**2**^	**Adjusted model**^**3**^	**BIC**
	**OR (95% CI)**	**OR (95% CI)**	**OR (95% CI)**	
**Prenatal BMI**				2323.777**
Underweight	0.7 (0.48, 1.02)	0.72 (0.49, 1.06)		
Normal	ref	ref	ref	
Overweight	1.32 (0.97, 1.8)	1.33 (0.97, 1.81)		
Obese	1.88 (1.16, 3.06)	1.84 (1.13, 2.99)		
**Interpersonal violence exposure**				2321.619**
No exposure	ref	ref	ref	
Exposure	0.85 (0.25, 2.91)		0.94 (0.27, 3.25)	
**Prenatal depression**				2320.494**
No depression	ref	ref	ref	
Depression	1.44 (0.77, 2.69)		1.58 (0.84, 2.98)	
**Insurance status**				2326.671*
Private	ref	ref	ref	
Public	1.0 (0.76, 1.33)		1.07 (0.8, 1.42)	
Uninsured	0.83 (0.5, 1.37)		0.88 (0.53, 1.45)	

*OR* odds ratio, *CI* confidence interval

^1^Unadjusted for confounders

^2^Adjusted for maternal age and insurance status

^3^Adjusted for maternal age

^*^Lowest BIC presented is the adjusted model

^**^Lowest BIC presented is the crude model

^a^Insufficient cell count

**Table 6:** AANHOPI Odds Ratios: Filipino

	**Unadjusted model**^**1**^	**Adjusted model**^**2**^	**Adjusted model**^**3**^	**BIC**
	**OR (95% CI)**	**OR (95% CI)**	**OR (95% CI)**	
**Prenatal BMI**				1786.028**
Underweight	0.84 (0.41, 1.74)	0.92 (0.44, 1.9)		
Normal	ref	ref	ref	
Overweight	1.81 (1.38, 2.37)	1.83 (1.4, 2.41)		
Obese	2.16 (1.58, 2.94)	2.17 (1.59, 2.97)		
**Interpersonal violence exposure**				1803.809**
No exposure	ref	ref	ref	
Exposure	0.79 (0.3, 2.07)		0.92 (0.34, 2.44)	
**Prenatal depression**				1804.053**
No depression	ref	ref	ref	
Depression	0.99 (0.58, 1.67)		1.08 (0.63, 1.85)	
**Insurance status**				1804.357*
Private	ref	ref	ref	
Public	1.1 (0.83, 1.47)		1.19 (0.89, 1.59)	
Uninsured	1.13 (0.7, 1.83)		1.19 (0.73, 1.93)	

*OR* odds ratio, *CI* confidence interval

^1^Unadjusted for confounders

^2^Adjusted for maternal age and insurance status

^3^Adjusted for maternal age

^*^Lowest BIC presented is the adjusted model

^**^Lowest BIC presented is the crude model

^a^Insufficient cell count

**Table 7:** AANHOPI Odds Ratios: Japanese

	**Unadjusted model**^**1**^	**Adjusted model**^**2**^	**BIC**
	**OR (95% CI)**	**OR (95% CI)**	
**Prenatal BMI**			360.9874^**^
Underweight	0.44 (0.1, 1.89)	0.44 (0.1, 1.88)	
Normal	ref	ref	
Overweight	1.55 (0.73, 3.32)	1.58 (0.74, 3.38)	
Obese	8.51 (4.02, 18.01)	9.06 (4.24, 19.4)	
**Interpersonal violence exposure**			379.1353^**^
No exposure	ref	ref	
Exposure	4.18 (0.37, 46.87)	4.58 (0.4, 52.15)	
**Prenatal depression**			378.2126^**^
No depression	ref	ref	
Depression	2.46 (0.78, 7.74)	2.44 (0.77, 7.71)	
**Insurance status**			
Private	ref	ref	
Public	0.72 (0.28, 1.88)	EMPTY	
Uninsured	1.21 (0.35, 4.21)	EMPTY	

*OR* odds ratio, *CI* confidence interval

^1^Unadjusted for confounders

^2^Adjusted for insurance status

^**^Lowest BIC presented is the crude model

^a^Insufficient cell count

**Table 8:** AANHOPI Odds Ratios: Native Hawaiian/Other Pacific Islander

	**Unadjusted model**^**1**^	**Adjusted model**^**2**^	**Adjusted model**^**3**^	**BIC**
	**OR (95% CI)**	**OR (95% CI)**	**OR (95% CI)**	
**Prenatal BMI**				75.14295**
Underweight	EMPTY	EMPTY		
Normal	ref	ref	ref	
Overweight	EMPTY	EMPTY		
Obese	3.07 (0.62, 15.08)	3.01 (0.57, 15.92)		
**Interpersonal violence exposure**				85.70298**
No exposure	ref	ref	ref	
Exposure	0.53 (0.05, 5.08)		0.92 (0.11, 7.96)	
**Prenatal depression**				82.50423**
No depression	ref	ref	ref	
Depression	3.67 (0.96, 14.05)		2.78 (0.68, 11.34)	
**Insurance status**				86.05976**
Private	ref	ref	ref	
Public	4.5 (0.93, 21.99)		4.1 (0.82, 20.43)	
Uninsured	2.25 (0.19, 27.31)		2.32 (0.19, 28.25)	

*OR* odds ratio, *CI* confidence interval

^1^Unadjusted for confounders

^2^Adjusted for maternal age and insurance status

^3^Adjusted for maternal age

^**^Lowest BIC presented is the crude model

^a^Insufficient cell count

**Table 9:** AANHOPI Odds Ratios: Other Asian

	**Unadjusted model**^**1**^	**Adjusted model**^**2**^	**Adjusted model**^**3**^	**BIC**
	**OR (95% CI)**	**OR (95% CI)**	**OR (95% CI)**	
**Prenatal BMI**				9028.086*
Underweight	0.65 (0.48, 0.89)	0.68 (0.5, 0.93)		
Normal	ref	ref	ref	
Overweight	1.62 (1.44, 1.83)	1.64 (1.45, 1.85)		
Obese	2.04 (1.77, 2.35)	2.12 (1.84, 2.45)		
**Interpersonal violence exposure**				9158.099*
No exposure	ref	ref	ref	
Exposure	1.17 (0.73, 1.89)		1.04 (0.65, 1.67)	
**Prenatal depression**				9151.39*
No depression	ref	ref	ref	
Depression	1.34 (1.02, 1.76)		1.47 (1.12, 1.94)	
**Insurance status**				9149.36*
Private	ref	ref	ref	
Public	0.85 (0.74, 0.98)		0.97 (0.84, 1.12)	
Uninsured	0.97 (0.79, 1.18)		1.11 (0.91, 1.36)	

*OR*: odds ratio, *CI* confidence interval

^1^Unadjusted for confounders

^2^Adjusted for maternal age and insurance status

^3^Adjusted for maternal age

^*^Lowest BIC presented is the adjusted model

^**^Lowest BIC presented is the crude model

^a^Insufficient cell count

In several instances, crude models exhibited lower BIC values compared to adjusted models (Tables 4, 5, 6, 7, 8 and 9). This suggests that the addition of covariates from a causal framework did not substantially improve the model’s explanatory power or fit. It is possible that the variables used for adjustment (e.g., maternal age, insurance status) introduced additional complexity without significantly altering the observed associations. The findings revealed notable variability in GDM risk factors across ANHOPI subgroups. Obesity emerged as a consistent and strong predictor of GDM across all groups, while other factors such as interpersonal violence exposure and prenatal depression demonstrated limited or subgroup-specific effects.

## Discussion

This study undertook a novel analysis of disaggregated PRAMS data to illustrate the association between race and ethnicity and GDM risk, uncovering several critical findings that demonstrate a significantly higher risk for GDM among Asian and NHOPI individuals as a group and different rates of risk factors between Asian and NHOPI ethnicities.

The results emphasize the sociodemographic differences unique to Asian and NHOPI individuals that contribute to their elevated GDM risk. Specifically, Chinese, Filipino, and Other Asian groups were found to be at an increased risk for GDM compared to Native Hawaiian individuals, with each group exhibiting distinct risk factors. It is notable that despite NHOPI individuals having a high prevalence of pre-pregnancy obesity, one of the greatest risk factors for GDM, Asian people had a higher risk for GDM in this sample. Unlike most prior studies investigating sociodemographic or genetic risk factors, which often exclude Asian and NHOPI populations due to small sample sizes or fail to disaggregate Asian and NHOPI ethnicities [51], this study included a comprehensive analysis of all Asian and NHOPI subgroups to reveal disparities that are typically masked by data aggregation [52]. GDM diagnosis was significantly associated with maternal ethnicity, suggesting that this factor may warrant further investigation to understand the underlying causes.

Regarding the overall analytic sample, the findings of this study highlight significant disparities in GDM prevalence and associated risk factors across racial and ethnic groups, emphasizing the complex interplay of sociodemographic, clinical, and behavioral determinants. Native Hawaiian/Other Pacific Islander and Native individuals exhibited the highest prevalence and odds of GDM compared to White individuals. Elevated BMI, particularly obesity, emerged as a critical driver of GDM risk across all groups, underscoring the need for targeted interventions addressing pre-pregnancy weight [24, 53–55]. Additionally, the association between prenatal depression and increased GDM risk highlights the importance of integrating mental health care into prenatal care to mitigate adverse outcomes [56]. Despite significant differences in sociodemographic characteristics, such as insurance type, education, and interpersonal violence exposure, these factors demonstrated varying degrees of influence on GDM risk. These differences may reflect structural inequities, such as limited access to preventive care and socioeconomic disadvantages, particularly among Native and Black populations, who also had high rates of public insurance use [57–60]. Elevated BMI, particularly obesity, further exacerbated GDM risk, also disproportionately affecting Black and Native mothers, where obesity prevalence was highest. Mental health disparities also contributed to GDM risk, with the highest prenatal depression prevalence observed among Multiracial individuals. These findings highlight the dual need for culturally responsive care that addresses both modifiable clinical risk factors, such as BMI and mental health, and broader structural inequities that underlie the disproportionate burden of GDM among minority groups [22, 61–63].

Our results confirm the higher prevalence of GDM among Asian and NHOPI individuals, consistent with prior studies indicating elevated risks in these populations compared to White individuals to contextualize this finding [9, 22, 30, 51, 64]. The elevated prevalence of GDM among Asian and NHOPI individuals is likely multifactorial, influenced by pre-pregnancy BMI, sociocultural factors, and potential genetic predispositions that may reflect different metabolic profiles, including a predisposition to insulin resistance at lower BMI thresholds. These findings emphasize the importance of disaggregating Asian and NHOPI subgroups in analyses, as substantial variation exists within this diverse population. [23, 60, 64–70]. However, while there is limited data on the complex interaction between modifiable and non-modifiable risk factors, the stark difference in GDM risk found between Asian and NHOPI and non-Asian and NHOPI individuals, and observed heterogeneity between Asian and NHOPI ethnicities, suggest that Asian and NHOPI race and ethnicity are both risk factors for GDM due to a combination of both body size and habitus, and sociocultural factors [23, 60, 64–70]. Several sociodemographic and behavioral factors significantly influenced GDM prevalence across racial and ethnic groups. The disaggregated analysis of Asian and NHOPI subgroups revealed notable heterogeneity. For instance, Filipino individuals exhibited higher rates of GDM, and prenatal depression compared to other subgroups, suggesting the need for tailored interventions. Conversely, Chinese and Japanese individuals had a higher prevalence of normal BMI yet still experienced elevated GDM rates, suggesting that BMI alone does not fully explain GDM risk in these populations. There is some evidence that East Asian people (i.e., people from China or Japan) tend to develop type 2 diabetes and other metabolic conditions, such as insulin resistance, at lower body mass indexes (BMIs) compared to other ethnic groups [71]. This may be due in part to a propensity for greater visceral fat accumulation despite appearing lean [71]. The higher rates of GDM among individuals classified as “Other Asian” underscore the need for improved data collection to better identify and address disparities within smaller subgroups. Future research should investigate lifestyle factors, dietary patterns, and culturally specific stressors contributing to these differences.

That psychosocial factors, prenatal depression and IPV exposure, and GDM status were not linked among Asian and NHOPI individuals in this study is somewhat surprising given prior studies and meta-analysis consistently documented the association between the two [9, 23, 72, 73]. Cultural factors may influence the utilization of mental health services with language barriers making it difficult for some Asian and NHOPI individuals to access healthcare services, along with mental health stigma and “shame or the loss of face” associated with mental health disorders and the lack of culturally competent providers and resources to meet diverse racial and ethnic needs [74–76]. There may also be cultural variations in the way anxiety and depression are expressed and self-reported, with Asian populations having more somatic symptoms of depression that are often not captured by screening measures including the brief PHQ measure used in PRAMS [77]. These factors may potentially explain the lower rates of prenatal depression prevalence reported in the Asian and NHOPI population compared to the other racial and ethnic groups in this study.

In 2004, a World Health Organization (WHO) consultation suggested that the current BMI cut-offs were not appropriate for the Asian population and lower BMI cut-offs for elevated BMI categorized as overweight (25–29.9) or falling into the obesity category (≥ 30) as several studies suggested that many cases of diabetes in Asian individuals occur at lower BMI levels [53]. It is unknown if these lower cut-offs would also apply to Native Hawaiian/Pacific Islanders [53]. A study investigating current BMI thresholds found that a BMI screening cut-off of 30 kg/m^2^ would identify 56.3% of Black individuals with diabetes in pregnancy, 32% of white individuals, 35% of South Asian individuals, and only 13% of East Asian individuals [54]. The ‘risk equivalent’, or “comparable to 30 kg/m^2^ in white women”, threshold for South and East Asian individuals was approximately 21 kg/m^2^ [54]. Thus, a race and ethnicity blind standard applying to patients, to better understand GDM risk, would not be effective. There is a unique BMI cutoff for Pacific Islanders known as Polynesian Pacific Islanders [78]. However, this standard is usually used specifically for Pacific Islanders living in Pacific Island nations [78]. Maternal nativities need to be considered if a unique BMI standard is to be implemented for Asian and Native Hawaiians/Other Pacific Islanders [78].

These data suggest that variation in GDM risk between disaggregated Asian and NHOPI ethnicities needs to be further investigated [52]. By specifically focusing on GDM in Asian and NHOPI from a large study sample, this study provides new insights into the existing literature regarding the interplay of race/ethnicity and sociodemographic factors in the disparities of GDM, as well as contributing risk factors in specific Asian and NHOPI ethnic populations. To better inform clinical practices, GDM diagnosis and management, and health equity within GDM, it is essential that future studies continue to include and potentially oversample Asian and NHOPI groups to be able to disaggregate between Asian and NHOPI ethnicities, and clarify risk factors unique to the diverse Asian and NHOPI population [13, 79].

### Strengths and limitations

This study has several notable strengths. The PRAMS data’s extensive coverage, inclusion of diverse maternal and child health indicators, and standardized data collection methodology provide a strong foundation for investigating maternal and child health in the US [80, 81]. Specifically, this study utilized seven years of national data, including disaggregated Asian and NHOPI ethnicity groups, to enable a detailed and robust analysis within this population. The responses for race and ethnicity were self-reported by participants, strengthening the accuracy of the racial/ethnic group data [80, 81]. Also, the selection of potential risk factors was determined with a hypothesis-driven approach and included factors beyond those well-documented in the current literature, to allow for a comprehensive analysis [2, 4, 65]. In terms of statistical analyses, the application of directed acyclic graphs (DAGs) and the Bayesian Information Criterion (BIC) strengthened the causal inferences drawn from the data and resulted in robust, parsimonious final models for both the overall analytic sample and Asian and NHOPI subsample [82, 83].

Another strength of this study is the disaggregation of Asian and NHOPI ethnicities. The disaggregated descriptive statistics of Asian and NHOPI ethnicities from this study revealed differences in the distribution of key social determinants of health between ethnicities, that would have been concealed with data aggregation and aims to contribute to the push for disaggregated data [52, 79].

This study should also be reviewed in the context of a few limitations, first relating to the utilization of PRAMS data. The PRAMS dataset consists of retrospective, cross-sectional data and survey answers may have been influenced by recall bias; as exposure variables are self-reported, there is the possibility of perception bias and nondifferential misclassification, an error in classification regardless of exposure or health outcome status [84]. The reliance on self-reported data may introduce reporting bias, particularly for sensitive variables such as interpersonal violence and smoking [85–88]. The response rate threshold for public data release is relatively low (55%), likely due to varying response rates by state. As data collected in 2020 was included, some of the low response rate may be explained by the COVID-19 pandemic [89]. However, a disparity exists among vulnerable populations such as Hispanic individuals, those with less than a high school education, and racial minority groups, resulting in a small sample of Asian and NHOPI respondents for the study’s data analysis [90]. PRAMS respondents are contacted by address data on birth certificates resulting in relatively low selection and response rates among non-English speaking groups and the vulnerable populations [80]. Additionally, the comparison of pre-gestational diabetes rates to rates of GDM by race/ethnicity group was not feasible, as there was no way to separate Type II diabetes, an acquired disease, and Type I diabetes, within the pre-gestational diabetes variable. Second, the Asian and NHOPI subsample created for this analysis was reduced in size due to missing values and potential lost observations as the survey did not offer an ethnicity option for Pacific Islander ethnicities other than “Native Hawaiian/Other Pacific Islander”, potentially masking differences between Asian and Pacific Islander subpopulations. The “Other Asian” group could include diverse groups of people such as Korean, Vietnamese, Hmong, Laotian, etc. and there was no option for individuals identifying as multiethnic. Third, there was also a lack of specific standardized variables that may be risk factors for GDM diagnosis such as family history, immigration status, sexual orientation, and gender identity [39]. The self-reported data also does not allow for further analysis of clinical data such as precise measurement of glycemic control, medication adherence, and specific lifestyle modifications [51].

## Conclusions

This analysis of 2016 to 2022 PRAMS data illustrated the significant variation of GDM predictors between races and Asian and NHOPI ethnic groups as well as highlighting the increased odds of GDM diagnosis among Asian and NHOPI people. Using disaggregated Asian and NHOPI subsample data, the descriptive statistics of this study also revealed differences in the distribution of risk factors of GDM, such as BMI, maternal education, and insurance status, between Asian and NHOPI ethnicities. The findings of this study emphasize the need for further research regarding sociodemographic and cultural risk factors within diverse Asian and NHOPI subpopulations and the potential benefits of culturally inclusive or adapted GDM prevention strategies across diverse ethnic groups.

## Data Availability

PRAMS data are available for request by researchers at: https://www.cdc.gov/prams/prams-data/researchers.htm.

## Abbreviations

CDC: Centers for Disease Control and Prevention
PRAMS: Pregnancy Risk Assessment Monitoring System
Asian and NHOPI: Asian and Native Hawaiian/Other Pacific Islander
NHPI: Native Hawaiian Pacific Islander
NHOPI: Native Hawaiian/Other Pacific Islander
GDM: Gestational Diabetes Mellitus
T2DM: Type 2 Diabetes Mellitus
HBP: High Blood Pressure
BMI: Body Mass Index
SCHIP/CHIP: State Children’s Health Insurance Program/Children’s Health Insurance Program
IHS: Indian Healthcare Service
TRICARE: Healthcare program for active service members, retirees, and their dependents
WHO: World Health Organization
